# Supplementary material for: Unveiling Niaprazine’s Potential: Behavioral Insights into a Re-Emerging Anxiolytic Agent
Source: Biomedicines. 2024 Sep 12;12(9):2087. doi: 10.3390/biomedicines12092087 (PMC11428487; doi:10.3390/biomedicines12092087)
Supplement: Supplementary file 1 [file biomedicines-12-02087-s001.zip › biomedicines-3137616-supplementary.pdf]

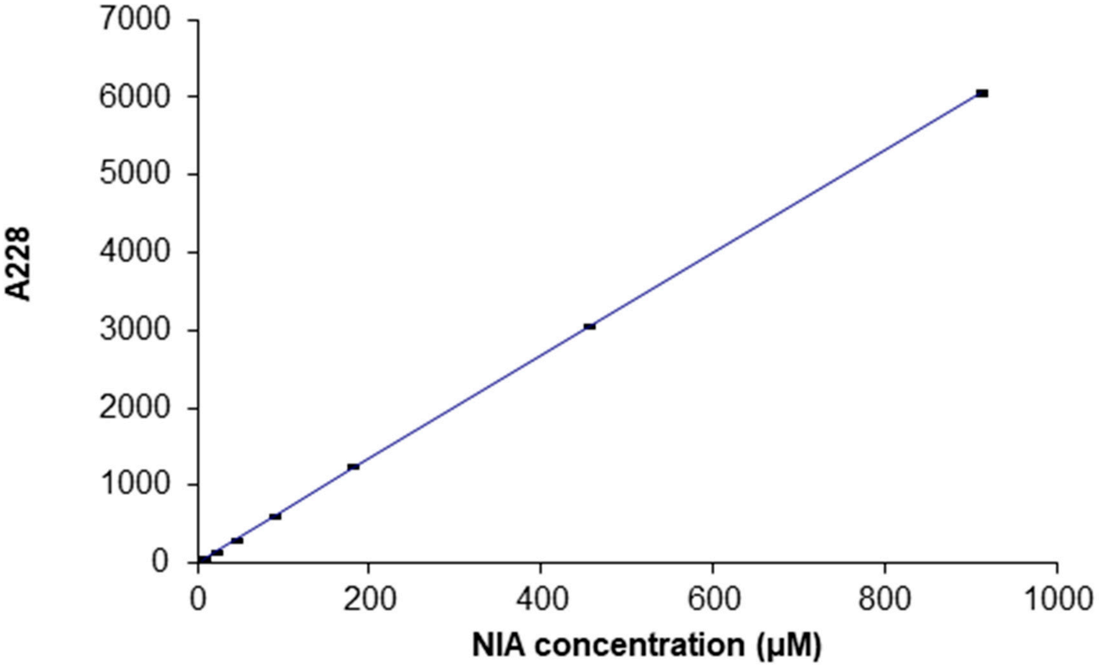

Figure S1. Assurance of stability of drinking solution. The plot shows the linearity of NIA peak areas in the chromatograms registered at 228 nm wavelength against NIA concentrations (values are reported as means ± SD, n = 3).

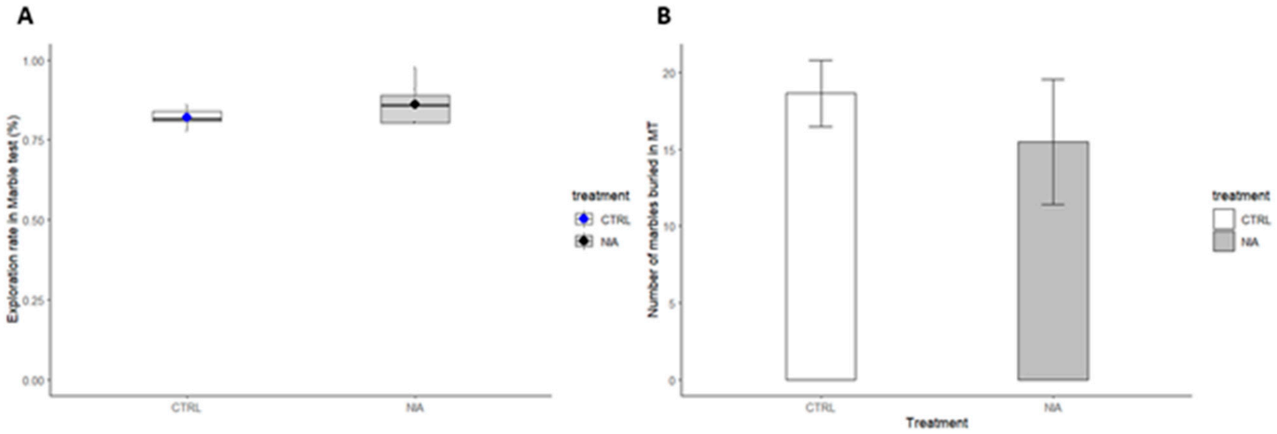

Figure S2. Box plots show the exploration rate in the control and NIA group (A). The exploration rate is expressed as the percentage of arenas visited during the test. The white box represents the control group with the mean indicated by a blue dot, and the grey box represents the NIA group with the mean indicated by a black dot. Bar plots represent the number of marbles buried during MT, with the white bar for control and the grey bar for NIA (B). Data are expressed as mean ± SEM. Statistical analysis was performed using the two-tailed Student’s t-test.
